# Microbially Induced Corrosion: A Hidden Risk in Industrial Accidents

**DOI:** 10.1021/acsomega.5c09790

**Published:** 2026-03-19

**Authors:** Tomáš Vítěz, Luboš Kotek, Michal Černý, Nikola Hanišáková, Monika Vítězová, Petr Trávníček

**Affiliations:** † Department of Agricultural, Food and Environmental Engineering, 309613 Mendel University in Brno, Zemědělská 1,Brno 613 00, Czech Republic; ‡ Department of Production Systems and Virtual Reality, 48274 Brno University of Technology, Technická 2896/2, Brno 616 69, Czech Republic; § Department of Experimental Biology, Section of Microbiology, Faculty of Science, 117204 Masaryk University, Kamenice 753/5, Brno 625 00, Czech

## Abstract

The phenomenon of microbiologically induced corrosion (MIC) has been known for more than 100 years, but its role as a cause of accidents in the process industry is still somewhat underestimated. Microbiologically induced corrosion is often associated with anaerobic environments, where microorganisms such as sulfate-reducing bacteria or methanogenic archaea are significantly involved in accelerating corrosion of metallic materials. This study aims to assess the significance of microbiologically induced corrosion in industrial accidents, identify facilities and conditions most at risk, and propose effective prevention strategies. Analysis of publicly available accident databases, together with expert assessment of operating conditions, suggests that MIC might be associated with roughly 10–20% of corrosion-related incidents; however, this range should be interpreted cautiously due to substantial limitations in reporting and confirmation practices. The presence of anaerobic microorganisms and biofilms substantially increases the likelihood of MIC, underscoring the need for systematic monitoring of microbial communities and environmental conditions to improve preventive measures and enhance plant safety. However, the analysis is limited by the incomplete and heterogeneous reporting of corrosion mechanisms in publicly accessible accident databases. Consequently, the estimated contribution of MIC should be interpreted with caution, as many events lack sufficient microbiological or environmental detail to allow definitive classification.

## Graphical abstract

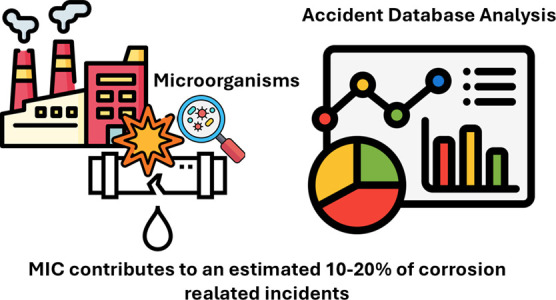

## 1 Introduction

Corrosion generates substantial financial losses in industrial facilities due to both preventive activities (maintenance, protective measures, planned replacement of degraded parts) and the consequences of corrosion-related accidents (production interruptions, equipment replacement, reputational damage). In addition, accidents can cause not only financial damage but also personal injury or environmental damage. According to the 2024 eMARS database, roughly 10% of all accidents involving Seveso-regulated equipment are linked to corrosion. Corrosion is also one of the important attributes of the physical aging of technology, which has received considerable attention not only in the European Union.^1^

It has been reported that in 2013, the global financial loss due to corrosion was around US$2.5 trillion.^2^ The mechanisms of corrosion that have been described so far are numerous (chemical, electrochemical, galvanic, etc.). One intensively studied mechanism is microbiologically induced corrosion (MIC), also referred to as microbial corrosion, biocorrosion, microbially influenced corrosion, or biodegradation.^3^ MIC has been recognized for more than a century, with the earliest reference from Garrett in 1891. MIC is estimated to cause about 20% of all corrosion damage,^4^ corresponding to more than $250 billion annually in the United States,^5^ particularly affecting oil extraction, water distribution, and power generation.^6^

MIC has become a widely studied topic, with 2060 publications listed in Web of Science, dominated by authors from China (804 publications), the USA (453 publications), Australia (147 publications), and Canada (124 publications). Publication output increased from 40 papers in 2012 to 204 in 2023.

Industrial accident databases are essential for analyzing incidents and preventing their recurrence; in the EU, the most commonly used include eMARS and France’s ARIA system. A preliminary review showed that MIC is rarely identified as an accident cause, despite being a well-known risk factor for pipelines, storage tanks, and heat exchangers.

The aim of this paper is to examine this issue in greater detail, specifically by analyzing available records of corrosion-related accidents with special attention to the role of MIC. To achieve this objective, the following steps were undertaken:

- Define a method for collecting and analyzing records of industrial accidents caused by corrosion
- Estimate the number of accidents caused by MIC based on available records
- Conduct an analysis of accidents associated with MIC
- Discuss the results of the analysis

MICs generated under anaerobic conditions can be considered a more serious problem than MICs generated under aerobic conditions for several reasons. These include the difficulty in detecting and controlling degraded material. This work therefore focuses on anaerobic MIC, typically classified as chemical MIC (caused by corrosive microbial metabolites such as acids or H_2_S) or electrical MIC. Chemical MIC accelerates corrosion by altering the environment, whereas electrical MIC involves direct electron transfer between microbial cells and the metal surface, often mediated by cytochromes.

Indirect electrical MIC involves extracellular electron transfer, typically through redox cycling of metabolites. Most MIC processes require microorganisms to be in direct contact with the metal surface within a biofilm. Planktonic micro-organisms, which may be freely present in the stored/transported medium, are usually not involved in MIC. Biofilm formation is therefore essential for MIC, with key anaerobic contributors including sulfate-reducing, nitrate-reducing, acid-producing and metal-oxidizing bacteria, as well as methanogenic archaea.

## 2 Methods

### 2.1 Information Sources

Information on incidents where microbial corrosion was the cause was taken from publicly available databases. These were the following information sources:

- Major Accident Reporting System (eMARS)
- Pipeline and Hazardous Materials Safety Administration Database (PHMSA database)
- National Transportation Safety Board Database (NTSB database)

#### 2.1.1 eMARS Database

Accidents were searched for in eMARS^7^ using search keywords. The keywords “Corrosion” or “Corroded” were used in the eMARS database search. Records attributed to accidents involving equipment covered by the Seveso I, II, and III Directives were considered relevant. Additional search criteria required that the causative factor be identified as “Corrosion”. In total of 107 items were identified. The following data were analyzed: year of event, industry type, event type, affected equipment, contained substance and corrosion type. First, cases explicitly mentioning MIC were identified. For the remaining cases, an expert assessment was made to determine whether MIC was likely based on available environmental and technical conditions.

#### 2.1.2 PHMSA Database

Searches of PHMSA’s database^8^ were limited to “pipeline” facilities. The database contained 113 records in total. Only events where corrosion was explicitly stated as the technical cause were included (repetition of “explicitly stated cause”). This filtering resulted in 25 identified events.

#### 2.1.3 NTSB Database

The search of the NTSB database^9^ was also limited to “pipeline” facilities, focusing on incidents from 1985 onward. A total of 81 events were searched in this way. Only events where corrosion was explicitly identified as the cause were considered (repetition again highlighted).

### 2.2 Expert Estimate

Records identified in the eMARS database were subsequently evaluated by an expert to determine whether the prevailing environmental and operational conditions indicated a probable presence of microbiologically induced corrosion (MIC). This evaluation was based on the available information regarding key environmental parameters. The expert’s decision-making process is illustrated in a decision diagram (Figure 1).

**Figure 1:**
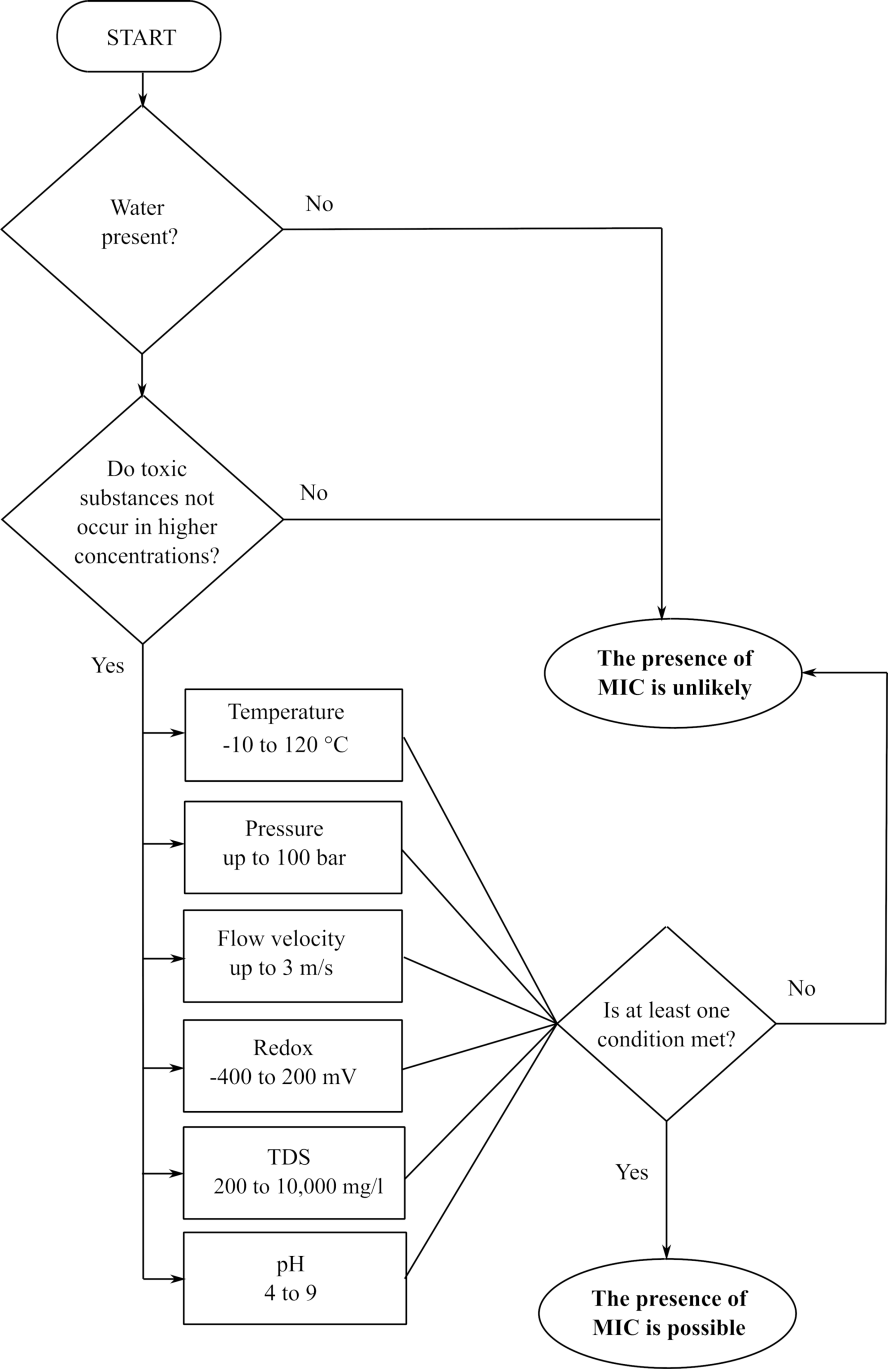
Decision diagram for assessing the likelihood of microbiologically induced corrosion (MIC) based on environmental conditions. The flowchart evaluates the presence of water, concentration of toxic substances, and key operational parameters (temperature, pressure, flow velocity, redox potential, total dissolved solids, and pH) to determine whether MIC is possible or unlikely.

Due to limited detail in some records, only two qualitative confidence levels were defined:

- Unlikely
  corrosion is reported, but
  environmental conditions are inconsistent with MIC development,
  or an alternative corrosion mechanism is clearly dominant, such as mechanical fatigue or purely chemical corrosion.
- Possible
  the database record, investigation report, or official documentation explicitly states that microbiologically induced corrosion was the cause of the event AND
  the expert review found no contradictory environmental or technical evidence that would rule out the presence of MIC.
  the database record identifies corrosion as the cause, but MIC is not explicitly mentioned, AND
  environmental or operational conditions described in the record are compatible with MIC processes, based on expert assessment (e.g., presence of moisture, stagnation, anaerobic zones, sulfides, organic load).

The key assessment parameters are explained in more detail in Chapter 4. Incidents were classified as confirmed if the database records or investigation reports indicated that the event was MIC-related and this was not contradicted by expert judgment.

## 3 Mechanisms of Microbiologically Induced Corrosion (MIC)

Microbial Induced Corrosion (MIC) is a form of material degradation caused by the metabolic activities of microorganisms. This corrosion can occur in both aerobic and anaerobic environments, driven by the complex interactions between microbes and metal surfaces. While planktonic (free-floating) microorganisms generally pose little risk to metal integrity, the real threat arises when these organisms attach to surfaces and form biofilms.

Biofilms play a central role in MIC by creating highly structured and heterogeneous microenvironments on metal surfaces. Microorganisms within biofilms form distinct metabolic zones depending on the availability of electron donors and acceptors. Hydrogen (H_2_) released from metallic iron or produced by fermentative bacteria fuels denitrifiers, sulfate reducers, methanogens, and acetogens, while heterotrophs and sulfide oxidizers remove oxygen and establish anaerobic conditions near the metal surface. Electroactive methanogens and Fe^3+^-reducing bacteria can directly extract electrons from Fe^0^ through multihaem cytochromes or conductive minerals, generating Fe^2+^ that iron oxidizers convert into Fe^3+^ oxides. These oxides subsequently serve as electron acceptors for Fe^3+^ reducers. The diffusion and sequential consumption of electron acceptors (O_2_, NO_3_^–^, SO_4_^2–^) create vertical stratification within the biofilm. This structure enables both indirect electron transfer via corrosive metabolites and direct electron transfer via conductive nanostructures and outer-membrane cytochromes, making biofilms a powerful driver of corrosion.^10,11^

Under aerobic conditions, Fe^2+^-oxidizing and Mn^2+^-oxidizing bacteria produce Fe­(III) and Mn^4+^ oxides that act as strong oxidants of Fe^0^, accelerating corrosion.^12,13^ Acid-producing bacteria such as *Acetobacter* spp. and *Acidithiobacillus* further enhance metal dissolution by generating organic and inorganic acids. For example, *Acetobacter* converts ethanol to acetic acid, which promotes pitting corrosion of steel and copper alloys.^14^

*Acidithiobacillus* produces sulfuric acid during thiosulfate oxidation, which significantly accelerates metal corrosion.^15^ Although stainless steel is generally protected by passive oxide films, these layers can be disrupted by biofilms. Bacteria such as *Bacillus subtilis* and acids within extracellular polymeric substances (EPS), including gluconic acid, can locally reduce pH and trigger corrosion.^16^

Under anaerobic conditions, oxidants such as nitrate, sulfate, and CO_2_ do not react spontaneously with Fe^0^; microbial catalysis is required. Anaerobic microorganisms promote Fe^0^ oxidation through corrosive metabolites, H_2_-mediated electron transfer, direct electron uptake from the metal surface, or electron shuttling via redox-active organic molecules.^10^

## 4 Key Parameters Affecting the Growth of Microorganisms

Several conditions must be met for microbiologically induced corrosion to occur, including the presence of microorganisms, availability of water and nutrients, suitable pH, temperature and salinity, and appropriate material properties. Flow characteristics, oxygen levels, and the chemistry of the transported medium also influence microbial activity, and the material–environment interface plays a critical role.

### 4.1 Nutrients

Nutrient availability is a key factor for the growth and metabolism of microorganisms. In environments with limited availability of nutrients (e.g., carbon, nitrogen, phosphorus), microorganisms may slow down their growth, enter a dormant state, or produce endospores for survival. Under anaerobic conditions, nutrient availability may be even more limited, affecting the efficiency of metabolic pathways.

### 4.2 Presence of Water

A dry environment greatly limits microbial growth because most microorganisms require moisture to survive and multiply. Water supports essential biological processes, including metabolism, nutrient transport, waste removal, structural stability, and osmotic regulation. Water serves as a medium in which chemical reactions take place and allows the dissolution of ions and molecules, which is crucial for the growth and function of bacteria.

When pipelines or storage tanks are partially or fully buried, soil properties strongly influence corrosion risk. The movement of ions through the electrolyte, which in this case is the soil, is necessary for corrosion to occur, and therefore factors that increase the electrical conductivity of the soil usually also increase the risk of corrosion. These factors include, for example, higher soil moisture, insufficient drainage or high salt content. All these factors contribute to an increased risk of corrosion.

### 4.3 Acidity and Alkalinity of the Environment

Most corrosion-related microorganisms tolerate a pH range of 5–10, but extreme pH disrupts protein stability, membrane permeability, and osmotic regulation. Despite external pH fluctuations, microorganisms maintain a near-neutral cytoplasmic pH to preserve essential metabolic functions. In acidophilic bacteria, the cytoplasmic pH is approximately 6.0, while in alkaline bacteria it ranges from 7.2–8.7.^17^

Tran et al.^18^ investigated how pH affects MIC caused by sulfate-reducing bacteria (SRB Their results show that SRB increased the pH from 4 to 7.5 within 5 days. Iron concentrations were highest at pH 4, reaching values up to three times higher than at pH 7.4.^18^ This suggests that at lower pH the corrosion process proceeds significantly faster than at neutral pH. Like abiotic corrosion.

### 4.4 Temperature

Environmental temperature has a fundamental influence on the activity of anaerobic microorganisms, as it affects their metabolism and growth rate. Each microorganism has an optimal temperature range in which it reaches its highest activity, and temperature limits at which it can survive, but its activity is limited. The range of temperature extremes that microorganisms can survive ranges from −20 °C (*Colwellia psychrerythraea*, *Methanogenium frigidum*) to 130 °C (*Geogemma barossii*, which can multiply at 121 °C).^19^ We can therefore assume that at temperatures above 121 °C, the environment can be considered unsuitable for the growth and multiplication of microorganisms.

At higher temperatures (above 80–90 °C), common, nonextremophilic microorganisms will experience protein denaturation and structural breakdown. It can therefore be said that a temperature in the technology above 80 °C can be considered sufficient to inactivate microorganisms that contribute to MIC. However, in the case of endospores, even such high temperatures may not be sufficient to inactivate them, and they may be activated when suitable conditions for life arise. Conversely, low temperatures below 5 °C slow down the metabolism of microorganisms and limit the biochemical reactions that are necessary for growth and reproduction. Higher temperatures also increase the release of metals such as vanadium, chromium, strontium, and cobalt from water pipes. These metals pose both health risks and contribute to MIC development.^20^

### 4.5 Pressure

Microorganisms exhibit a wide range of adaptations to high and low pressure. This makes them able to survive in environments with extreme conditions, such as deep oceans or high mountain areas. The pressures commonly used in technology do not pose a direct risk to the life of microorganisms. *Thermococcus piezophilus*, a thermophilic archaeon is able to survive up to 125 MPa.^21^ On the contrary, *Deinococcus aetherius* survived for a year in conditions that simulated the low pressure in space (10^–13^–10^–10^ MPa).^22^ It can therefore be stated that the pressure value in technologies will not affect the activity of microorganisms, but rather other factors, such as the solubility of gases or the state of water.

### 4.6 Salinity

High salinity alters microbial community structure by causing cell dehydration and disrupting metabolic functions. It also affects enzyme activity, water activity, and environmental pH, which together influence microbial activity. High salinity is also associated with disorders in osmoregulation.^19^ The total salinity of the environment is associated with the occurrence of a wide range of different ions, Na^+^, Cl^–^, SO_4_^2–^, Ca_2_^+^, and Mg_2_^+^.^23^ It can be stated that salt concentrations above 1.7 M NaCl (11.68 g/L) influence the inhibition of the process. However, there are microorganisms that are able to survive concentrations up to 4 M NaCl (46.72 g/L) or 5 M MgCl_2_.^24^

Corrosion in brine pipelines can occur when salt concentrations still permit microbial activity. Salt caverns containing sulfate-rich seawater show high microbial activity, and MIC risk is particularly elevated in hydrogen storage systems.^25^ This can explain some cases of brine pipeline degradation and subsequent accidental liquid leakage, e.g.^26^

### 4.7 Shear Stress

Shear stress, which is exerted on a biofilm by fluid movement, such as in water or in a pipe, can affect biofilm stability. High values of shear stress can damage or remove the biofilm, affect the growth of microorganisms, and prevent biofilm formation.^27^

### 4.8 Biofilm

Biofilm formation will play a key role in MIC, as it promotes the colonization of metal surfaces by microorganisms, creates suitable conditions for their metabolism and accelerates electrochemical reactions that lead to metal degradation. Biofilm is a specific environment that allows many different groups of microorganisms to live and protect themselves from external influences. A number of processes will take place in the biofilm that will influence the kinetics of corrosion.^28^

### 4.9 Others

One factor that can affect the MIC on the outside of buried pipelines is the chemical and microbial population of the soil. Two studies have identified high levels of sulfide in the soil and linked them to a high risk of aggressive corrosion due to the presence of SRB. Avoiding locations with high levels of sulfide in the soil may be one way to inhibit the growth of SRB biofilms.^29,30^ High concentrations of metals, toxic gases such as H_2_S or NH_3_, and high redox potential can inhibit microbial growth. These factors disrupt anaerobic microbial communities and reduce MIC activity.

## 5 Analysis of Accidents

Using the methodological procedure described above, 107 records meeting the criteria in Section 2.1 were identified in which corrosion was listed as a contributing factor. Of these 107 records, 48 were classified—based on the procedure in Section 2.2—as events in which MIC is possible. In one case, the database entry suggested that the operator considered MIC a possible contributing factor, although this was not formally confirmed.

The case involved a “near-miss” at an oil refinery in an off-site unit, where a rarely used 20-year-old pipeline—with no documented maintenance—was operated during one of the processes.

### 5.1 Industrial Sector

The industry breakdown was taken from the eMARS database. Among the 107 corrosion-related events, 34% occurred in the “Petrochemical/Oil Refineries”. The “General Chemicals” category then came second with 32%, see Figure 2 When only events where MIC was considered a co-factor were included, the share of incidents in the petrochemical/oil refinery sector rose to 42% (Figure 3).

**Figure 2:**
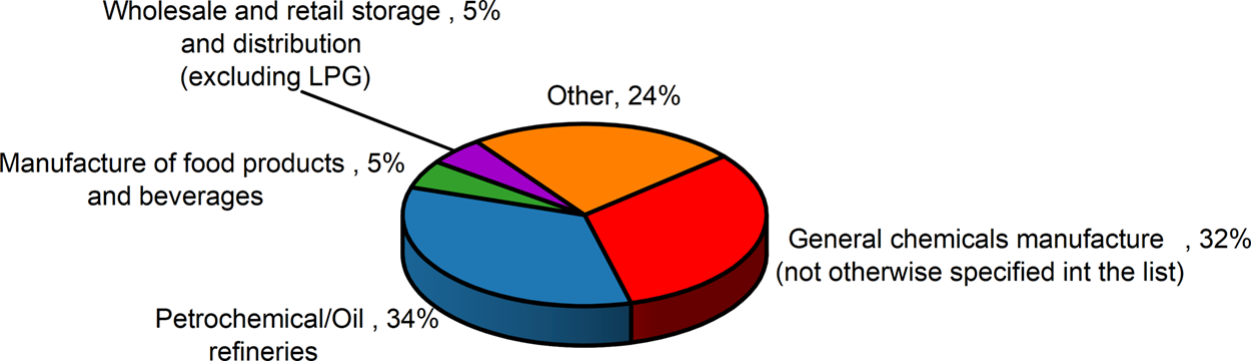
All studied industrial sectors (aggregated data from the eMars database).

**Figure 3:**
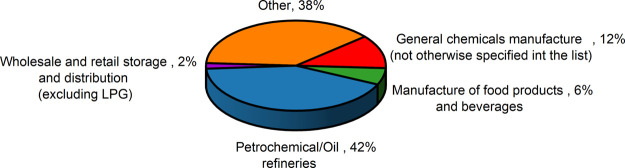
Industrial sectors in which MIC is considered to have occurred (aggregated data from the eMars database).

### 5.2 Type of Corroded Equipment

Equipment affected by corrosion was classified into eight categories: Valve, Pipeline, Reactor, Sensor, Heat Exchanger, Tank, and Other. In cases where the corroded component was part of a larger unit, the event was assigned to the parent equipment group. For example, pipe corrosion inside a heat exchanger was classified under “Heat Exchanger,” and flange corrosion associated with a storage tank was categorized as “Storage Tank”, etc.

The category “Valves” includes events related to valve corrosion. There was a total of 10 cases. These involved valves that were part of a storage tank, heat exchangers, drain valves or valves that were part of a piping system. Overwhelmingly, the top event was a substance leak. The leakage occurred, for example, due to tightening (thread corrosion, corrosion of the spring mechanism), spontaneous loosening of the manual valve cover.

A total of 6 records were identified that could be categorized as “Reactor”. These included failures due to corrosion of flange connections, lids, or rupturing discs. Due to the environmental conditions in the reactors, no events with the potential for MIC were categorized as such.

The category “Sensors” mainly included events where corrosion caused a measurement or control failure, which subsequently led to the realization of a peak event. These were a total of three cases. In two cases it was a pressure sensor failure and in one case it was a level measurement failure. In addition, the “Sensors” category included a case where a hazardous chemical leak occurred due to corrosion leaks. This was probably corrosion of the sensor at the connection to the pipe.

A total of 8 events has been categorized under “Heat Exchanger”. These were air or water coolers, reaction product coolers, evaporators. The “Other” category included events that were associated with corrosion of storage units (steel drums), filter equipment supports (collapse was followed by fire).

Figure 4A,B present the distribution of events across equipment categories for all corrosion cases and for MIC-related cases. In both data sets, “Pipeline” account for approximately 50% of events, followed by “Tank”. For MIC specifically, tanks represent 32% of cases compared to 19% in the full data set, meaning that pipelines and tanks together account for 86% of all MIC-related events.

**Figure 4:**
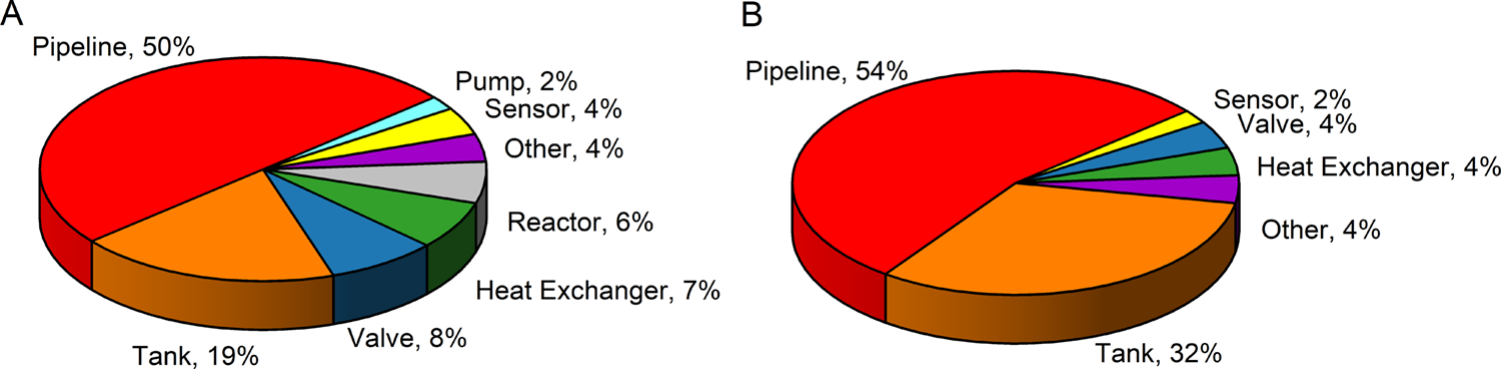
Proportion of each type of equipment, A – whole sample; B – MIC‘s sample (aggregated data from eMars database).

Approximately 20% of all recorded accidents occurred off-site. Corrosion of pipelines buried below ground level was identified in about 5% of cases. Among the records where MIC was considered possible, 34% occurred off-site. In the single case involving a buried pipeline, MIC was considered a plausible contributing factor.

For storage tanks, corrosion was located at the tank bottom in 35% of cases, on the tank shell in 15%, and at the shell–floor interface in 20%. The true proportion at the shell–floor interface is likely higher due to missing detail in several records. Other cases involved corrosion of structural supports, which in one instance led to complete tank collapse after the supports lost their mechanical integrity. If we consider only cases where the conditions for MIC exist, the percentages are similar (given the sensitivity of the data to changes in relatively small samples).

### 5.3 Detained Chemical Substance

A total of 80 hazardous chemicals and mixtures were identified in the database as present during the incidents. For analytical purposes, these substances were grouped into 14 chemical categories based on their predominant components, and when appropriate, further subdivided by their physical state. For example, hydrocarbons were split into “Liquid hydrocarbons” and “Gaseous hydrocarbons”. Other categories included “Hydrogen and mixtures”, “Hydrogen sulfides and mixtures”, “Acids”, and similar groups.

For clarity, only five chemical groups are shown in Figure 5A,B, with hydrocarbons clearly dominating both data sets. In the subset where MIC was considered possible, hydrocarbons accounted for more than half of all events. This is consistent with the ability of many anaerobic microorganisms to utilize hydrocarbons as substrates, producing organic acids, alcohols, methane, and carbon dioxide.

**Figure 5:**
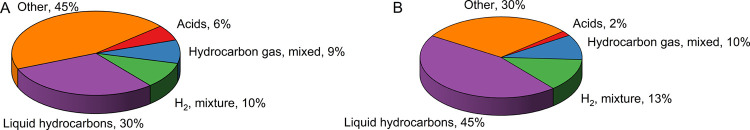
Kind of detained chemical substance A – whole sample; B - MIC‘s sample (aggregated data from eMars database).

### 5.4 Accidents on Long-Distance Pipelines

The results so far indicate that pipelines represent a critical corrosion-susceptible component in the process industry. To explore this further, accident data from the PHMSA and NTSB databases were analyzed. These databases provide more detailed incident investigation reports than eMARS, and PHMSA’s Accident Investigation Division routinely performs microbiological testing as part of its investigations.^31^

Twenty-five corrosion-related pipeline incidents were identified in the PHMSA database for the years 2004–2015. Pipeline ages ranged from 8 to 71 years. Most incidents (64%) involved pipelines transporting liquid hydrocarbons such as gasoline, crude oil, or jet fuel, while the remaining cases involved natural gas pipelines. PHMSA investigators confirmed MIC in nine cases, representing 36% of all corrosion-related incidents, which is considerably higher than in the eMARS data set. In eMARS, only one MIC case was reported among 26 pipeline-related events. An overview of PHMSA-confirmed MIC cases is provided in the (Table 1).

**Table 1:** Basic Information on Accidents with Confirmed MIC (PHSMA Database)

year of accident	top event	financial loss (ths. USD)	year the component that failed was installed	transported substance	location of corrosion	accident pressure estimate (bar)
2014	leakage	976	1952	diesel	internal	2.8–3.4
2013	leakage	13,844	1979	crude oil	internal	1.0
2013	leakage	3693	1978	crude oil	internal	27.6
2011	leakage	2953	1947	gasoline	external	2.8
2010	leakage	16	1976	crude oil	internal	10.3
2010	leakage	715	1947	natural gas	internal	49.6
2010	leakage	57	1950	natural gas	external	38.6
2009	leakage	249	1981	natural gas	internal	50.0
2005	leakage	17	1958	crude oil	internal	39.4

The NTSB database contained 13 incidents in which corrosion was identified as a contributing factor, occurring between 1986 and 2021. These events generally had more severe consequences than those recorded in the PHMSA database. In several cases, escaped natural gas or flammable vapors from liquid hydrocarbons ignited, causing not only financial losses but also fatalities and injuries among nearby occupants. The investigation reports show that out of 13 incidents, only 1 case was corrosion caused by microbiological activity. This was a natural gas leak and subsequent fire that occurred in 2000. The pipeline was commissioned in the mid-1970s.

### 5.5 Accidents on Underground Gas Storage Well

Microbial-induced corrosion (MIC) represents a significant concern for underground gas storage (UGS) facilities, particularly regarding well casings that are exposed to groundwater and microbial activity. A substantial body of research has underscored the pivotal role of microbial processes in corrosion mechanisms, underscoring the necessity for comprehensive risk assessments in UGS operations.

Research^32^ has indicated that underground storage facilities, such as aquifers and depleted reservoirs, may function as large-scale bioreactors for biomethane production through power-to-gas technology. However, the presence of microorganisms in these environments, including sulfate-reducing bacteria, poses a risk of microbial corrosion. Specifically, stored hydrogen may undergo microbial-induced changes, leading to the formation of hydrogen sulfide, which can degrade gas quality and accelerate corrosion processes.

Investigation of Aliso Canyon SS-25 well eruption underscores the significance of MIC. The research revealed that the corrosion patches were randomly distributed along the casing’s circumference, thereby ruling out traditional corrosion mechanisms such as crevice or galvanic corrosion. The observed striated grooves and tunnels on the corroded surface indicated MIC as the primary corrosion mechanism. The incident, which resulted in an uncontrolled hydrocarbon release for 111 days, was attributed to external microbial corrosion facilitated by groundwater intrusion and the presence of CO_2_ as a microbial nutrient.^33^

Additionally, CO_2_ plays a crucial role in subsurface corrosion processes. While CO_2_ is relatively stable in deep, high-temperature, and high-pressure environments, its interaction with microbial communities can lead to secondary transformations contributing to MIC. The geochemical characteristics of CO_2_ in natural gas reservoirs vary, with microbial activity influencing its composition and concentration. This highlights the necessity of further research into microbial influences on CO_2_ behavior and its implications for corrosion in UGS infrastructure.^34^

In summary, MIC presents a substantial threat to the integrity of underground gas storage wells, necessitating comprehensive geological, chemical, and microbiological evaluations. Future hydrogen storage projects must consider microbial interactions to prevent gas composition alterations and structural degradation due to MIC. Addressing these risks through improved monitoring, material selection, and microbial control strategies is crucial for ensuring the long-term safety and reliability of UGS systems.

## 6 Representative Case Studies

To strengthen the interpretation of the analysis results, several representative accident events were selected. In their detailed examination, particular attention was paid to the industrial sector in which the event occurred, the type of transported or stored substance, and the specific part of the technological system where the failure took place. Special emphasis was also placed on the methods used to identify microbiologically induced corrosion (MIC).

### 6.1 High-Pressure Natural Gas Transmission Pipeline (Carlsbad, New Mexico, USA)

The accident occurred on Saturday, August 19, 2000, near Carlsbad, New Mexico, USA.^35^ The pipeline was part of the El Paso Natural Gas Company transmission system. It consisted of an underground steel pipeline, API 5L grade B, with a diameter of 30 in. (762 mm) and a wall thickness of 0.375 in. (9.5 mm). It transported nonodorized natural gas at a pressure of 675 psig (approximately 46.5 bar). The pipeline was buried underground except for a section crossing the Pecos River, where it was supported by a steel bridge.

The technical cause of the accident was wall thinning of the steel pipeline due to corrosion. The investigation concluded that the corrosion was likely caused by a combination of microbial activity, moisture, chlorides, oxygen, carbon dioxide, and hydrogen sulfide inside the pipeline. The root cause of the event was identified as the failure of El Paso Natural Gas Company’s internal corrosion control program, which did not prevent, detect, or manage internal corrosion. Ineffective inspections by regulatory authorities also contributed, as they failed to identify deficiencies in the company’s corrosion control program.

The analysis of the failed pipeline was conducted in several phases, focusing on identifying the causes of internal wall degradation. After the accident, samples of corrosion products and deposits were collected from the internal surface of the pipeline and subjected to laboratory examination. Analyses were performed both in El Paso Natural Gas laboratories and specialized external facilities. Chemical analysis revealed extremely high concentrations of chlorides (up to 333,000 ppm), iron, sulfates, and hydrogen sulfide, indicating an aggressive corrosive environment.

Microbiological tests confirmed the presence of sulfate-reducing bacteria (SRB) and other anaerobic microorganisms typical of MIC. Samples were cultured on selective media and analyzed using microscopy and standard biochemical tests. However, the investigation report did not specify the exact technical standards used for microbial detection. Metallurgical assessment employed optical and electron microscopy, revealing sharp-edged pitting corrosion.

Based on the report, the investigation attributed corrosion to microbial activity for several reasons:

a. **Chemical Analysis of Deposits**
  Samples from the pipeline interior contained extremely high concentrations of chlorides (up to 333,000 ppm), iron, sulfides, and other ions. These substances are typical products of microbial metabolism, particularly SRB, which produce hydrogen sulfide (H_2_S) and cause localized pitting corrosion.
b. **Microbiological Test Results**
  Laboratory cultivation confirmed the presence of SRB, acid-producing bacteria, and both aerobic and anaerobic microorganisms. These microbes are known to form biofilms and create corrosive environments inside pipelines.
c. **Metallurgical Analysis of the Pipeline**
  At the failure site, sharp-edged pitting corrosion and significant wall thinning were observed. This morphology corresponds to MIC-related damage rather than uniform chemical corrosion.
d. **Operating Conditions of the Pipeline**
  The failure occurred at a low point in the pipeline where liquids (water, condensate) accumulated. Stagnant liquids created an anaerobic environment ideal for microbial growth. The pipeline transported nonodorized natural gas containing moisture, CO_2_, H_2_S, and chlorides, all of which promote microbial activity.

### 6.2 Transport of Liquid Hydrocarbons in Above-Ground Pipeline (United Kingdom)

The accident occurred on an onshore pipeline at a refinery facility in the United Kingdom.^36^ The exact date is not specified in the source. The pipeline was a steel pipe with a diameter of 6 in. (153 mm), used to transport a mixture of crude oil and seawater from a water separator to a slop oil tank. The pipeline was installed above ground. As a result of the failure, approximately 450 m^3^ of a mixture of liquid hydrocarbons and water was released.

According to the report, the technical cause was a combination of internal and external corrosion. Internal corrosion was significantly accelerated by the presence of seawater and stagnant conditions, creating an environment conducive to microbiologically induced corrosion (MIC). Microbial analyses revealed a substantial microbial load, including sulfate-reducing bacteria (SRB) and sulfate-reducing archaea (SRA), which are typical agents of MIC.

External corrosion developed due to atmospheric conditions, particularly on the underside of the pipeline where moisture accumulated. The protective coating was damaged, allowing gradual moisture ingress and formation of corrosion products. At the failure site, critical wall loss was observed, reducing thickness to 0.7 mm, with a perforation measuring 73 × 55 mm, resulting in loss of containment.

To identify total bacterial load, SRB, and SRA, quantitative polymerase chain reaction (qPCR) was employed. The report does not explicitly describe the detailed procedure but refers to NACE TM0212-2018 as a recommended standard for MIC detection and microbial load monitoring.

Based on the report, the investigation attributed corrosion to microbial activity for several reasons:

a. **Microbiological Analysis Results (qPCR)**
  qPCR confirmed significant microbial presence, including SRB and SRA, which are strongly associated with MIC.
b. **Operating Conditions**
  The pipeline was dormant (not in use), and the mixture of oil and seawater was stagnant. MIC typically occurs in stagnant or low-flow systems.
c. **Physical Evidence of Corrosion**
  Laser scanning and visual inspection revealed localized internal corrosion, perforations, and sediment deposits (pp. 5–7). The presence of a thick sediment layer (approximately 30 mm) inside the pipeline suggests biofilm formation and stagnation (pp. 6).

### 6.3 Stainless Steel Instrumentation Tubing (Type 316L)

The failure occurred in instrumentation tubing made of stainless-steel type 316L, part of a system leading to a control analyzer.^37^ The tubes had an outer diameter of 14 inch (approximately 6.35 mm). The first tube failed after an unspecified period of operation, while the replacement tube ruptured only 8 days after installation, indicating extremely high biological activity within the system.

Cooling water from a vortex cooler flowed through the tubing at approximately 4 °C; however, the temperature at the failure point was higher. The system was characterized by low flow and likely stagnation prior to commissioning (following hydrostatic testing), conditions that are ideal for the development of microbiologically induced corrosion (MIC). The inner surface of the tubes was covered with a brown film, suggesting insufficient water purity and the presence of nutrients for bacteria.

The investigation confirmed that the failure was caused by MIC induced by sulfate-reducing bacteria (SRB). These microorganisms create acidic environments beneath biofilms (e.g., sulfuric acid production), which aggressively attack metal surfaces. Evidence included localized pits with bulbous morphology, tunnelling and linear pitting, surface etching indicative of acid attack, and Scanning Electron Microscopy (SEM)/ Energy-Dispersive X-ray Spectroscopy (EDS) analysis detecting sulfur and chlorine at damage sites. Both tubes met the chemical composition requirements for 316L stainless steel and exhibited no microstructural defects, ruling out material flaws. The report does not specify whether a recognized standard was applied for MIC determination.

Consequences included water leakage. The first tube failed at a bend, while the second exhibited multiple pinhole perforations along its length. Recommendations included cleaning and disinfecting the system, inspecting other low-flow components, and checking stored materials for possible MIC damage.

The investigation attributed corrosion to microbial activity based on the following:

a. **Operating Conditions**
  Low flow (low-flow line) and stagnation of water.
b. **Visual Evidence of Damage**
  Damage morphology consistent with MIC: bulbous/undercut pits, isolated pinhole leaks, linear pitting, and tunnelling on the inner surface.
c. **Chemical and Material Analysis Results**
  SEM revealed etched surfaces (acid attack). EDS detected sulfur (linked to SRB acid production) and chlorine. No material defects were found (316L stainless steel met specifications, no sensitization).

### 6.4 Other Extraordinary Events

The scientific literature reports numerous other extraordinary events where MIC can be assumed as the technical cause. For example, an incident in the UK in 2012 ^3^ involved severe wall thinning in a pipeline transporting a multiphase mixture (gas, oil, and water). The thinning was detected in time, preventing leakage. The standard MPN method had been used since 2002; later, qPCR revealed high counts of SRB, SRA, and methanogens on the pipeline surface, indicating that MPN underestimated the actual microbial load.

Starosvetsky et al.^38^ describe three additional MIC-related events in the petrochemical industry. In the first case, carbon steel piping in a refinery heat exchanger failed after about 18 months of operation. Massive fouling of tubes, covers, and joints with rust occurred, along with severe localized corrosion. Deposits up to 6 cm thick formed, beneath which pitting corrosion reached depths of around 10 mm. The cause was activity of iron-oxidizing bacteria, which accelerated corrosion product formation and led to fouling and equipment damage.

The second case involved corrosion of floating roofs in storage tanks at a petrochemical facility. The roofs were made of aluminum alloy Al 5052. Corrosion occurred during a three-week hydrostatic test. White corrosion products appeared on the underside of the roofs, and severe localized corrosion rapidly developed, leading to complete perforation. The cause was contamination of test water with microorganisms (heterotrophic bacteria, SRB, and fungi), whose biofilm significantly accelerated corrosion.

The third case concerned a diesel engine cooling system. Aluminum components (pipe elbows, thermostat covers, and parts of the radiator made of alloy A03560) were exposed to coolant consisting of 20% ethylene glycol and water. After some time, leaks appeared due to pitting corrosion of these parts. The cause was MIC, supported by the presence of heterotrophic bacteria in the coolant and reduced inhibitor effectiveness after dilution with water. MIC identification procedures used in these cases are summarized in the (Table 2).

**Table 2:** Summary of Procedures for Detecting MIC^a^

case	common methods	specific methods	key evidence of MIC
1. heat exchanger (carbon steel)	visual inspection; chemical analysis of water; EDS + SEM of corrosion products; microbiological cultivation (HAB, SRB, IOB)	morphological analysis of tubercles; staining of filamentous cells	identification of filamentous iron-oxidizing bacteria (*Sphaerotilus*) + typical tubercular deposits
2. floating roof (Al 5052)	visual inspection; chemical analysis of water; EDS of corrosion products; microbiological cultivation (HAB, SRB, fungi)	simulation tests (sterilized vs original water)	corrosion only in contaminated water, no corrosion in sterile water
3. cooling system (Al alloy)	visual inspection; analysis of coolant; microbiological analysis	long-term laboratory tests of HAB growth at different ethylene glycol concentrations; electrochemical tests (potentiodynamic curves)	pitting only in the presence of microorganisms, confirmed by HAB growth and electrochemical data

a HAB: Heterotrophic aerobic bacteria; SRB: sulfate-reducing bacteria; and IOB: iron-oxidizing bacteria.

## 7 Discussion of the Analysis Results

In the eMARS database, only one of 107 records explicitly identified MIC as the cause. In contrast, expert assessment indicated that 48 cases exhibited conditions suitable for MIC development. This highlights a substantial discrepancy between confirmed MIC cases and those with favorable conditions for MIC.For pipeline-related incidents, MIC was identified in 9 of 25 cases in the PHMSA database, compared to only 1 of 13 cases in eMARS and 1 of 13 in the NTSB database. These differences illustrate substantial variation between information sources.The main reasons for these discrepancies can generally be explained by the following factors:

- Differences in reporting systems among databases, which influence the outcome
- Industry practices or standards
- Measurement methods affecting the results

### 7.1 Differences in Reporting Systems

Differences in the number of records for pipeline-related accidents may be due to variations in incident reporting systems within organizations managing accident databases and their primary objectives. PHMSA, operating under the U.S. Department of Transportation, regulates and oversees oil, natural gas, and hazardous materials pipelines. It develops regulations, conducts inspections and audits, and collects mandatory incident reports rather than investigating accidents directly. Its reporting forms Form 7100 ^39^ and Form 7000 ^40^ explicitly list microbial corrosion as a technical cause, and its scope is narrower than that of the other two databases.

For example, the NTSB focuses on multiple areas. It is an independent federal agency whose primary task is investigating major transportation accidents—aviation, rail, road, marine, and pipeline. Its goal is to improve safety through recommendations rather than regulation, often concentrating on the most severe cases.

The eMARS database is managed by the Major Accident Hazards Bureau (MAHB), part of the Joint Research Centre (JRC) of the European Commission. Its primary purpose is to collect and share information on major accidents under the Seveso Directive (2012/18/EU) for learning purposes. It serves as a tool for regulators, operators, and experts to analyze causes, consequences, and recommendations. The database includes accidents from various process industry sectors and does not focus solely on pipeline transport of chemicals. Cause classification is more general, and MIC is not specifically highlighted. Reporting to eMARS is done by national authorities based on investigation reports. These authorities may not be experts in accident cause analysis and may not consider MIC information important, leading to potential distortions.

These differences suggest that the relative frequency of MIC-related accidents varies mainly due to inconsistencies in reporting systems. Analysis of the ARIA database supports this interpretation. Among 605 corrosion-related incidents, only 24 mentioned microbial corrosion, many originating from a single facility with likely continuous MIC monitoring. Overall, MIC frequency remained low. Like eMARS, ARIA collects data for learning purposes, but its scope extends beyond Seveso-related major accidents. Records come from diverse sources, including operators, authorities, emergency services, and media.

### 7.2 Industry Practices or Standards

Differences in reporting systems may explain the variation in MIC frequency among databases, but they do not fully account for the large discrepancy between the single MIC case reported in eMARS and the 48 cases identified by expert assessment in Chapter 2.2. A key factor is likely the reporting system, but contributing factors may include industry practices or standards. In the petrochemical industry, extraction, long-distance transport, or storage of liquid or gaseous hydrocarbons, MIC has long been recognized and extensively studied. This is reflected in the number of scientific publications focused on this sector. As shown in the representative studies in Chapter 6, MIC can also occur in facilities unrelated to hydrocarbon transport or storage. This suggests that MIC may receive insufficient attention in sectors outside petrochemical operations.

### 7.3 Measurement Methods

Microbiologically induced corrosion is a highly complex process, making it challenging to demonstrate conclusively. One common approach to identify microorganisms in a sample is through culture-based tests. However, the presence of a specific genus in culture does not automatically indicate that the technology is at risk of corrosion. A key factor affecting the reliability of results is correct sample collection and storage prior to analysis. Improper handling can introduce contamination and lead to false positives. Because the detection of corrosion-promoting microorganisms depends on multiple variables, supplementary data is essential for accurate assessment.

An illustrative example is the accident described in Chapter 6.4. The facility used culture tests as part of its corrosion management program. These tests did not identify ongoing MIC, which was only detected using other methods. Culture tests are problematic because they are limited to selected microbial groups. It is often overlooked that only 1–10% of microorganisms in natural or technical systems are culturable using standard laboratory methods. Without modern molecular biological techniques, it is difficult to describe the role of other metal-oxidizing or reducing microorganisms, archaea, and fungi. Therefore, the actual number of MIC cases may be higher than reported in accident databases.

## 8 Measures to Prevent MIC

### 8.1 Measures Taken at the Design Stage of the Installation

Preventive measures against the occurrence of MIC should be initiated at the technical design stage of the plant. These preventive strategies can be divided into groups:

- Selection of appropriate materials,
- the change of environmental condition (presence of water, gas/liquid phase composition, etc.),
- the change of the metal-environment interface (use of coatings, coatings, cathodic protection, etc. for both internal and external surfaces).

#### 8.1.1 Appropriate Materials

An important aspect of MIC prevention is the reduction of biofilm formation. This can be achieved in the design phase of the device by reducing the surface roughness of the material. This is because biofilms are generally less likely to form on surfaces that are smooth. In piping, this can be achieved, for example, by minimizing areas of high internal stress caused by material processing, such as annealing or heat treatment after welding, reducing the frequency of welds, or polishing welds.^30,41^ However, these measures can also be used in the design of other devices such as storage tanks.

Another strategy may be to choose an inherently safe design, i.e., choosing a metal material that is resistant to MIC. However, this strategy is proving to be problematic. For example, the authors^42^ conducted an experiment with galvanized steel. They assumed that zinc is toxic to sulfate-reducing bacteria and many other microorganisms. The results of the study showed that this did not prevent biofilm formation.

Nonferrous metals generally exhibit greater corrosion resistance compared to carbon steel, with titanium being a notable example. However, titanium’s cost—approximately 30 times higher than carbon steel—restricts its application to smaller equipment or specialized technologies. However, even for noble materials such as the aforementioned titanium, MIC has been described to occur in both aerobic^43^ and anaerobic environments.^44^ Similarly, MIC corrosion has also been observed in copper and its alloys.^45^

#### 8.1.2 The Change of Environmental Condition

The presence of water is a key factor in the development of microbiologically induced corrosion. Therefore, systems for controlling humidity and water content should be considered already at the design stage of the technology. Physical water-removal methods include gravity separators, centrifuges, demisters and filtration systems that separate water by density or mechanical barriers. Moisture can also be removed by cooling the gas below its dew point, causing water to condense and be eliminated from the system.

Chemical or physical methods include the use of dehydrating agents such as glycols (e.g., Triethylene glycol) that absorb water from the gas phase, or the application of chemical inhibitors that prevent moisture from condensing on the surface of the equipment. Other principles include membrane technologies, where gases and water are separated based on different permeability, and the application of vacuum systems to reduce the partial pressure of water. Monitoring systems can be integrated into the technology to track parameters relevant to MIC, such as microbial presence, humidity and changes in chemical composition. These mitigation methods are often used in combination. Effective moisture removal markedly reduces the likelihood of creating conditions favorable for microbial growth.

#### 8.1.3 The Change of the Metal-Environment Interface

Polymer coatings or liners can also be used to reduce MIC. Plastic liners are an effective barrier against corrosion, but problems arise when holes are present in the pipe that allow corrosive materials to migrate between the pipe material and the liner.

In the case of internal coatings, it is very difficult to apply them evenly, which affects their effectiveness. The occurrence of various damages, grooves and crevices in polymer coatings has been shown to favor the occurrence of corrosion. Some studies have shown preferential colonization of heterogeneous surfaces such as cracks, scratches or small holes.^46,47^

A strong bond between the coating and the material is also a fundamental prerequisite for coating effectiveness. If this bond is weak, the resulting space may become microbiologically contaminated and, in conjunction with increased humidity, growth may occur. Another problem is that the coating material itself can suffer from MIC. This is because some microorganisms can use the substances contained in the coating as a source of nutrients. This will subsequently cause a reduction in the protective ability of these coatings.^48^ A possible way is to develop and produce coatings with antimicrobial and anticorrosion properties.^49^

### 8.2 Measures Taken during Operation

Internal corrosion usually requires the presence of an electrolyte to complete the corrosion cell. This electrolyte is usually water or other water-based substances. In addition, certain chemicals are often necessary to initiate and sustain corrosion, such as carbon dioxide (CO_2_), which can form dilute acids, or sulfur, which can contribute to acid formation or support bacterial growth. Once these corrosive substances enter the pipe, they can cause permanent damage until they are removed or consumed by corrosion reactions.

In general, methods for influencing internal corrosion of technologies during operation include:

- physical methods (regular mechanical removal of biofilm),
- physicochemical methods (dehydration),
- chemical methods (buffering, application of corrosion inhibitors)

#### 8.2.1 Mechanical Removal of Biofilm

Mechanical cleaning tools are regularly used to remove and clean the internal surfaces of pipelines and to remove biofilms, impurities and water in which microbial activity could occur. Various principles are used, e.g. pigging, flushing, ultrasonic treatment. However, this approach does not prevent further biofilm formation, and in addition, it requires expensive ongoing maintenance. Once the surface of the technology is in contact with a suitable environment, biofilm can form within minutes.^50^

#### 8.2.2 Dehydration

If water is detected in the system, it must be removed promptly to prevent corrosion or microbiologically induced corrosion (MIC). Operators may apply a combination of physical, chemical, and other mitigation methods described in Chapter 5.1. The use of real-time moisture monitoring systems is also recommended, as these can help identify the source of water intrusion and enable rapid corrective action to prevent recurrence.

#### 8.2.3 Buffering

Buffers that modify the chemical composition of transported or stored fluids can be used to mitigate internal corrosion. Adding a mild alkaline buffer can reduce the corrosivity of water, and alkaline conditions are generally nonaggressive toward steel. However, buffering is often impractical for large-scale systems, where achieving uniform distribution is difficult and overall effectiveness is therefore limited.

#### 8.2.4 Corrosion Inhibitors

As mentioned earlier, microbiologically induced corrosion (MIC) is a type of corrosion that is accelerated or facilitated by the presence and activity of microorganisms. The aim of corrosion inhibitors is to reduce or prevent microbial activity that leads to corrosion. These can be, for example, biocides, surfactants, and others.

##### 8.2.4.1 Biocides

prevent the growth of microorganisms and the formation of biofilms, which are often responsible for the onset and progression of corrosion. Both oxidative and nonoxidative biocides are used in industry. Oxidative biocides include chlorine (chlorine compounds), which interact with cells and cause their death. However, oxidizing agents can also interact with technology materials and cause their corrosion. The second group of commonly used substances are nonoxidative biocides Tetrakis Hydroxymethyl Phosphonium Sulfate (THPS), glutaraldehyde, quaternary ammonium salts, isothiazolones, organobromines, oxazolidines, triazines and others.^51,52^ Benzalkonium chloride is also used in industry due to its broad-spectrum antimicrobial effects against bacteria, fungi and viruses.^53^ However, most biocides are very expensive to produce, toxic and difficult to decompose, which poses a serious threat to nature.

Currently, there is an intensive search for nontoxic, biodegradable natural chemicals that can be used as ecological corrosion inhibitors and biocides. The use of natural substances, such as plant extracts, for the treatment of MIC is considered environmentally friendly. Many plant oils and aqueous plant extracts have been shown to have inhibitory effects on yeasts, bacteria, archaea, and filamentous micromycetes.^54^ Unfortunately, the extraction of these substances is expensive.

##### 8.2.4.2 Surfactants

are another popular antibacterial agent. They are divided into five main classes according to their chemical composition: phospholipids, lipopeptides, glycolipids, polymeric compounds and neutral lipids. The adsorption of the functional group of biosurfactants onto the metal surface is the most important measure in the corrosion inhibition process. The adsorption ability of biosurfactants is related to their ability to agglomerate to form micelles and form a protective layer on the metal surface.^55^

##### 8.2.4.3 Others

A variety of emerging MIC mitigation strategies have been proposed, including essential oils, nanomaterials, specialized surface coatings, organic compounds, antiprotein films, and bacteriophages.^56,57^ However, large-scale industrial application of these methods is currently unrealistic due to limitations in required dosage, cost, efficiency, and safety. Moreover, the underlying mechanisms of corrosion inhibition for these alternative methods remain insufficiently understood, indicating the need for further research. Nitrate injection into oil fields was primarily carried out with the aim of reducing oil acidity. Based on experiments, it was found that the composition of the microbial community changes when nitrates are injected. There was a dramatic decrease in the presence of sulfate-reducing bacteria SRB,^58^ which will directly affect the formation of MIC.

## 9 Discussion

In the case of searching for information in the eMars database, out of a total of 107 records, only 2 cases explicitly stated that it was a MIC. However, the expert estimated that the technology had suitable conditions for the formation of MIC in a total of 48 cases. This is therefore a relatively large disproportion between confirmed cases of MIC and cases where these conditions were suitable for the formation of MIC.

This disproportion stands out when comparing the outputs from the eMars database and the PHMSA database, which focuses on events that occurred on pipelines for long-distance liquid transport. As stated in the text above, MIC was identified in a total of 9 cases out of 25. In contrast, in the case of the eMars database for pipeline transport, MIC was identified in only 1 case out of 26. In the NTSB database, it was 1 case out of 13. This means that there are also significant differences between individual information sources. The question therefore arises, what can cause these differences in outputs?

Two basic hypotheses can be put forward to explain these differences:

- MIC is underestimated
- Inaccurate measurements are being taken

### 9.1 MIC is Underestimated

The PHMSA organization directly states in its investigation reports that MIC tests are also performed as standard in the case of accidents. However, this cannot be said with complete certainty for other information sources. It is therefore possible that the role of MIC as a factor influencing accidents may be underestimated in the industry in general. From this point of view, it may be interesting to assess the outputs from the ARIA industrial accident database.^26^ This database is one of the largest publicly accessible databases of industrial accidents covering events from various types of human activity. It includes more than 50,000 records. A total of 605 records associated with corrosion of metal materials were identified here, which ultimately led to an accident in an industrial facility. Only in 25 cases was it mentioned in the records that MIC could have occurred. At the same time, however, these events very often repeat themselves in the same facilities. Therefore, there will be significantly fewer unique records from different facilities. It seems that some companies have a requirement in their standards to investigate the possibility of MIC, but some do not address this issue. This may indicate that MIC is underestimated in some companies and that the role of MIC in accidents may be greater than is thought.

### 9.2 Inaccurate Measurements are Being Taken

Culture-based tests are commonly used to detect microorganisms, but their results do not necessarily indicate a corrosion risk. Proper sampling and storage are essential, as errors may lead to contamination and false positives. Because many factors influence the detection of corrosion-related microorganisms, culture tests alone provide insufficient information.

Commercial test kits for soil, water, and corrosion deposits are widely used to screen for microbial activity. According to PHMSA records, MIC confirmation often relies on “Quick Kits” designed to detect aerobic and anaerobic heterotrophs, sulfate-reducing bacteria (SRB), and acid-producing microorganisms. Only in a few accidents, analyses were performed that would describe the multidisciplinary phenomenon of MIC based on the analyses listed above. An example is the NACE TM0194-2014 standard,^59^ which only addresses the above-described groups of microorganisms. Cultivation methods rely on selective media to isolate groups such as SRB or iron-oxidizing bacteria, but many environmental microorganisms are difficult or impossible to culture. Because typically only 1–10% of microorganisms are culturable, these methods provide an incomplete view of the microbial community. As a result, traditional cultivation methods often fail to capture the full diversity of microbial populations, leading to an incomplete understanding of microbial communities involved in microbial-induced corrosion (MIC). To overcome the limitations of culturing, molecular approaches such as 16S rRNA sequencing (for *Bacteria* and *Archaea*), internal transcribed spacer (ITS) sequencing (for *Fungi*), and quantitative polymerase chain reaction (qPCR) for gene quantification, have been widely applied. These molecular techniques provide broad microbial community profiles but cannot distinguish living from dead cells, which may lead to overestimation of the active population and misinterpretation of microbial activity. Transcriptomic techniques help overcome this limitation by identifying actively expressed genes, providing insight into real-time microbial activity. When integrated with genomic and metabolomic data, transcriptomics supports a multiomics approach that distinguishes active from dormant microorganisms and improves understanding of MIC-related processes.

Several rapid on-site methods are available for assessing microbial activity, including ATP photometry for biomass estimation, fluorescence microscopy for visualizing biofilms, and enzymatic assays such as hydrogenase and APS-reductase measurements to detect specific microbial functions like sulfate reduction. These rapid techniques, while not as comprehensive as molecular approaches, are valuable tools for real-time monitoring and can complement more in-depth analyses in industrial settings. The integration of molecular techniques, transcriptomics, and rapid field methods allows for a more complete and accurate assessment of microbial communities involved in MIC. By combining qualitative and quantitative data, these approaches provide deeper insights into the microbial dynamics, ultimately facilitating better management and mitigation of corrosion in industrial systems.^11^

To determine whether MIC contributed to an accident, multiple analyses are required to reconstruct the preincident environmental conditions. These include molecular methods, cultivation tests, elemental and mineralogical analyses, metallography, and microscopy.

## 10 Methods for Assessing MIC

The evaluation of microbiologically influenced corrosion (MIC) requires an integrated microbiological, chemical, and materials science approach. MIC results from microbial metabolism that alters electrochemical processes on material surfaces. Accurate identification and quantification of these interactions are essential for effective corrosion management in the energy, petrochemical, water treatment, and transportation sectors. Over recent decades, MIC assessment has progressed from traditional culture-based techniques and visual inspections to advanced molecular diagnostics capable of rapidly and precisely characterizing microbial communities. This development is reflected in international standards that define standardized procedures for MIC detection across industrial sectors. An example is the NACE TM0194-2014 standard^59^ and TM0212-2018 focus on visual inspection, surface sampling, and culture-based methods for determining microbial populations, complemented by electrochemical measurements (e.g., potential profiles). NACE TM0194-2014 emphasizes field monitoring using selective media for sulfate-reducing bacteria and other groups, while the newer NACE TM21465-2024 introduces molecular techniques (qPCR, 16S rRNA analysis) for rapid identification of microbial communities. ISO 11130 and ISO/DIS 21055 prioritize controlled laboratory testing using sterilized apparatus, defined inocula, and weight-loss measurements. ASTM C1894-22 focuses on concrete materials and uses simulated environments inoculated with microbial cultures to evaluate degradation. Overall, older standards rely primarily on culture-based techniques and visual assessment, while recent guidelines incorporate molecular diagnostics and standardized laboratory protocols to achieve higher accuracy and reproducibility (Table 3).

**Table 3:** Comparative Overview of Methodological Approaches in International Standards for MIC Assessment

standard	visual inspection	culture-based methods	molecular methods	electrochemical methods
NACE TM0106-2016	yes	yes	no	yes
NACE TM0212-2018	yes	yes	no	yes
NACE TM0194-2014	no	yes	no	no
NACE TM21465-2024	no	limited	yes	no
ISO 11130	no	yes	no	no
ISO/DIS 21055	no	yes	no	no
ASTM C1894–22	no	yes	no	no

## 11 Conclusion

This work proposes a simple procedure for identifying MIC-related incidents in industrial accident databases and introduces a method for expert estimation of the probable occurrence of MIC at affected facilities. Detailed analysis revealed a major discrepancy between records explicitly reporting MIC and those in which expert assessment indicated that MIC was likely involved. Significant differences in MIC occurrence across databases were observed, mainly due to variations in reporting systems and the differing objectives of the organizations maintaining these data sets. Explicitly reporting the type of corrosion involved could improve identification of MIC-related cases, provided that the personnel processing these records are adequately trained in recognizing MIC-related conditions.

Differences in industry practices and measurement methods may also influence reporting and lead to false negatives. Therefore, determining whether MIC contributed to an accident requires additional analyses to reconstruct preincident environmental conditions. These analyses should include molecular and cultivation techniques, as well as elemental, mineralogical, metallographic, and microscopic examinations. A major challenge in MIC assessment is the absence of a unified industrial standard defining sampling and analytical procedures. Existing international standards (e.g., NACE, ISO, ASTM) use varying approaches, from visual inspection and culture-based methods to electrochemical and molecular diagnostics. This methodological diversity reflects the evolution of MIC testing but also creates inconsistencies that hinder result comparability and effective mitigation. Developing an integrated and standardized protocol would improve reliability, enable cross-industry comparison, and support the implementation of best practices in MIC management.

Accident data show that pipelines and tanks containing liquid hydrocarbons are the most critical components, as these environments often support microbial growth. However, microbial presence in other equipment, including heat exchangers, shut-off valves, and detectors, should not be overlooked. Selected representative case studies indicate that MIC is not limited to equipment where liquid or gaseous hydrocarbons occur.
